# Clinical implications of gut microbiota and cytokine responses in coronavirus disease prognosis

**DOI:** 10.3389/fimmu.2023.1079277

**Published:** 2023-03-24

**Authors:** Hye Seong, Jun Hyoung Kim, Young-Hee Han, Ho Seong Seo, Hak Jun Hyun, Jin Gu Yoon, Eliel Nham, Ji Yun Noh, Hee Jin Cheong, Woo Joo Kim, Sooyeon Lim, Joon Young Song

**Affiliations:** ^1^ Department of Internal Medicine, Korea University College of Medicine, Seoul, Republic of Korea; ^2^ Asia Pacific Influenza Institute, Korea University College of Medicine, Seoul, Republic of Korea; ^3^ Vaccine Innovation Center - Korea University College of Medicine, Seoul, Republic of Korea; ^4^ Division of Infectious Diseases, Department of Internal Medicine, Chungbuk National University Hospital, Cheongju, Republic of Korea; ^5^ Department of Food and Nutrition, Chungbuk National University, Cheongju, Republic of Korea; ^6^ Research Division for Radiation Science, Korea Atomic Energy Research Institute, Jeongeup, Republic of Korea

**Keywords:** prognosis, gut microbiota, COVID-19, SARS-CoV-2, cytokine, host immune response, taxonomic markers, functional markers

## Abstract

**Objectives:**

Severe acute respiratory syndrome coronavirus 2 (SARS-CoV-2) infects gut luminal cells through the angiotensin-converting enzyme-2 receptor and disrupts the gut microbiome. We investigated whether the gut microbiome in the early stage of SARS-CoV-2 infection was associated with the prognosis of coronavirus disease (COVID-19).

**Methods:**

Thirty COVID-19 patients and 16 healthy controls were prospectively enrolled. Blood and stool samples and clinical details were collected on days 0 (enrollment), 7, 14, and 28. Participants were categorized into four groups by their clinical course.

**Results:**

Gut microbiota composition varied during the clinical course of COVID-19 and was closely associated with cytokine levels (*p*=0.003). A high abundance of the genus *Dialister* (linear discriminant analysis [LDA] effect size: 3.97856, *p*=0.004), species *Peptoniphilus lacrimalis* (LDA effect size: 4.00551, *p*=0.020), and *Anaerococcus prevotii* (LDA effect size: 4.00885, *p*=0.007) was associated with a good prognosis. Starch, sucrose, and galactose metabolism was highly activated in the gut microbiota of the poor prognosis group. Glucose-lowering diets, including whole grains, were positively correlated with a good prognosis.

**Conclusion:**

Gut microbiota may mediate the prognosis of COVID-19 by regulating cytokine responses and controlling glucose metabolism, which is implicated in the host immune response to SARS-CoV-2.

## 1 Introduction

Coronavirus disease (COVID-19) is a novel respiratory infectious disease caused by severe acute respiratory syndrome coronavirus 2 (SARS-CoV-2). During the early stage of the COVID-19 pandemic, most patients with COVID-19 presented with self-limiting mild respiratory illness, although 14% suffered from severe disease, and 2.45% of these patients died (1). Old age and underlying medical conditions are important risk factors for severe disease and poor prognosis. However, large individual variations are observed, irrespective of age and comorbidities. Thus, individual host factors must be better understood in addition to viral virulence factors.

SARS-CoV-2 enters human cells mainly *via* the angiotensin-converting enzyme 2 receptor (ACE2), which is present throughout the body, including in the respiratory and gastrointestinal tracts (2). Thus, patients with COVID-19 have prolonged fecal SARS-CoV-2 shedding, which causes gut inflammatory damage and microbial dysbiosis associated with disease severity (3). Together with the direct cytotoxic effect of SARS-CoV-2, three major mechanisms – dysregulation of the renin–angiotensin–aldosterone system, thrombo-inflammation, and cytokine dysregulation – contribute to disease progression (4). Therefore, gut microbiota possibly plays an important role in COVID-19 by influencing immune regulation and balancing cytokine production (2). In this aspect, fecal microbiota transplant (FMT) might restore the damaged gut microbiome and can be an additional therapeutic option (5). Clinical trials are ongoing to evaluate FMT’s impact on reducing the risk of disease progression in patients under standard COVID-19 treatment.

Few studies have evaluated the association between gut microbiota composition and the severity of COVID-19, and longitudinal data on gut microbial changes concerning disease prognosis are limited. This study aimed to evaluate the correlation between the gut microbiota and the prognosis of COVID-19 with regard to the host immune responses (cytokine release and antibody production) and metabolism. We investigated serial changes in gut microbiota and cytokines up to 4 weeks after symptom onset and analyzed their correlation with the prognosis of COVID-19.

## 2 Materials and methods

### 2.1 Study design and participants

In this prospective cohort study, 30 hospitalized patients with COVID-19 and 16 healthy controls were recruited from January 8, 2021, to September 9, 2021, at two tertiary hospitals in the Republic of Korea (Korea University Guro Hospital and Chungbuk National University Hospital). SARS-CoV-2 infection was confirmed using real-time (RT) polymerase chain reaction (PCR) of nasopharyngeal specimens. Patients who received probiotics within 4 weeks of symptom onset were excluded. Healthy individuals were enrolled as controls after excluding those who received any medications, including antibiotics, probiotics, laxatives, and motility drugs, that could affect the microbiome within 4 weeks prior to study enrolment. All controls tested negative for SARS-CoV-2 on nasopharyngeal RT-PCR and serological tests. Fecal and blood samples were collected serially on days 0 (enrolment), 7, 14, and 28. Patient data related to demographics, medications, chest radiography findings, laboratory results, and dietary records were obtained by two trained physicians. The analysis period was classified into week 1 (days 0–7), week 2 (days 8–14), week 3 (days 15–21), week 4 (days 22–28), week 5 (days 29–35), and week 6 (days 36–42) in the post-infection period.

Patients were assessed at enrolment based on the eight-category National Institute of Allergy and Infectious Disease Ordinal Scale score (NIAID-OS; **Supplementary Table 1**). Based on their clinical courses, patients were categorized into four groups: (A) recovery from mild COVID-19 (maintenance of an NIAID-OS of 4 during hospitalization); (B) improvement from moderate to mild COVID-19 (an improvement from an NIAID-OS of 5 to an NIAID-OS of 4); (C) improvement from severe to mild COVID-19 (an improvement from an NIAID-OS of 6 to an NIAID-OS of 4); and (D) deterioration (worsening from an NIAID-OS of 4 or 5 to an NIAID-OS of 7 or 8).

The study was approved by the Institutional Review Board of Korea University Guro Hospital (2020GR0570) and the Institutional Review Board of Chungbuk National University Hospital (2020-12-021). All participants provided informed consent. All procedures were performed according to the ethical standards of the institutional and/or national research committee and in accordance with the Declaration of Helsinki, 1964, and its later amendments or comparable ethical standards.

### 2.2 Sample collection and processing

Fecal samples were collected using Faecal Swab DNA Preservation and Transport Kits (Noble Bio, Hwaseong, Republic of Korea) containing nucleic acid preservation media. Fecal samples in the fecal swab transport medium were stored at −80°C. Blood samples were obtained by venipuncture and were collected in a serum-separating tube, which was subsequently centrifuged at 2500 rpm at −4°C for 10 min, and the serum-containing supernatant was pipetted into a clean plastic screw-cap vial and stored at −80°C.

### 2.3 Cytokine assay

We selected five cytokines that are known to influence the clinical course of COVID-19 (6, 7). Blood samples of patients with COVID-19 were tested, and a panel of cytokines, including interleukin (IL)-6, IL-10, tumor necrosis factor-alpha (TNF-α), interferon-gamma (IFN-γ), and IL-2, were quantified using the standardized Luminex xMAP system, which enabled multiplexed simultaneous quantification. This system uses spectrally addressed bead sets, each of which is conjugated with a specific capture monoclonal antibody for a given target molecule. The bead mixtures were analyzed using a Luminex 200 machine to quantify the signal per bead address and determine these analytes’ levels. Human Magnetic Luminex Assay (5 Plex) kits for individual assays were purchased from R&D Systems (Minneapolis, MN, USA).

### 2.4 Microbiological analysis

Total DNA was extracted using a PowerFecal^®^ Pro DNA kit (Qiagen, Germany) following the manufacturer’s instructions. At ChunLab, Inc. (Seoul, Republic of Korea), PCR amplification was performed using fusion primers that target the V3–V4 regions of the 16S rRNA gene, and mixed amplicons were pooled and sequenced using the Illumina MiSeq Sequencing System (Illumina, San Diego, CA, USA) according to the manufacturer’s instructions. The microbiological analysis process includes DNA extraction, PCR amplification, and sequencing of 16S rRNA and sequence data processing and analysis, which was performed using EzBioCloud 16S-based Microbiome Taxonomic Profiling and ChunLab’s bioinformatics cloud platform (described in the online **Supplementary Appendix**).

### 2.5 Nutritional surveys

The dietary intake survey of the participants was conducted using the food frequency questionnaire (FFQ) that was developed as part of the Korea National Health and Nutrition Examination Survey (KNHANES) (8, 9). The FFQ collects information regarding 112 food items, including rice, noodles and dumplings, bread and rice cakes, soups and stews, soybeans, eggs, meat and fish, vegetables, seaweed and potatoes, milk and dairy products, fruits, snacks, and alcoholic and non-alcoholic beverages. The FFQ has nine options for the frequency of consumption of each food (rarely, once a month, 2–3 times a month, once a week, 2–4 times a week, 5–6 times a week, once a day, twice a day, and three times a day), and three options for the portion size (small, medium, and large) compared to the standard amount.

### 2.6 Statistical analysis

All continuous variables are expressed as the median ± interquartile range, and categorical variables are presented as numbers (percentages). Non-parametric tests (paired *t*-test or Wilcoxon rank-sum test) were used to detect intergroup differences. The Kruskal–Wallis test was used to evaluate differences in the measured values among the four prognostic groups, and multiple comparisons were adjusted using the Bonferroni correction. The Spearman rank test was performed for the 112 food items and nutrient intake for the correlation analysis. Statistical analyses were performed using R version 4.1.2 (R Foundation for Statistical Computing, Vienna, Austria). Associations between the gut microbiota composition and serum inflammatory cytokine and antibody concentrations of the patients with COVID-19 were analyzed using the VEGAN, ggPLOT2 package in R. Principal component analysis of variance was conducted to identify the best set of variables that describe the community structure by fitting linear models to distance matrices and using a permutation test. Regarding the prognosis-stratified groups, five serum inflammatory cytokines were presented along the direction, and these were scaled by their correlation such that the weak predictors had shorter arrows than the strong predictors. The plotted cytokine arrows are depicted in the microbiome NMDS plot using the ggplot2 graph, which keeps the relative r2-scaled lengths of the arrows using VEGAN in the R package.

## 3 Results

### 3.1 Baseline characteristics of the four prognostic groups

Among the 30 patients with COVID-19, 5 were in group A (recovery from mild COVID-19), 9 were in group B (an improvement from moderate to mild COVID-19), 12 were in group C (an improvement from severe to mild COVID-19), and 4 were in group D (deterioration). As presented in **Table 1** and **Supplementary Table 2**, the median ages of the patients in groups A, B, C, D, and the control group were 62, 70, 63, 59, and 43.5 years, respectively. Two patients (one from group A and one from group B) withdrew from the study after their second follow-up, and one died after her third follow-up. Despite no statistically significant intergroup difference in the underlying medical conditions, the Charlson comorbidity index was rather high, and patients with end-stage renal disease (ESRD) were included in group A (*n* =2, 40%). Regarding the laboratory findings, the lactate dehydrogenase (LDH) level (A: 525.0 ± 225.0 vs. B: 695.0 ± 551.0 vs. C: 929.0 ± 248.0 vs. D: 818.5 ± 441.0, *p*=0.033) was lower in group A than in the others.

**Table 1:** Baseline characteristics of COVID-19 case and healthy control.

Characteristics	COVID-19 case (*n* = 30)				*P* value	Healthy control (*n* = 16)
	A (*n* = 5)	B (*n* = 9)	C (*n* = 12)	D (*n* = 4)		
**Age (years)**	62.0 ± 32.0	70.0 ± 21.0	63.0 ± 27.0	59.0 ± 35.0	0.466	43.5 ± 27.0
**Male sex (%)**	2 (40.0%)	7 (77.8%)	7 (58.3%)	3 (75.0%)	0.585	7 (43.8)
**BMI (kg/m^2^)**	23.3 ± 11.2	23.7 ± 6.7	25.4 ± 5.0	25.5 ± 8.6	0.581	25.4 ± 3.7
**Duration from symptom onset to enrollment (days)**	3.0 ± 6.0^a^	7.0 ± 6.0^a^	8.5 ± 7.0^a^	4.5 ± 4.0^a^	**0.028**	–
**ATB use within 1week. yes (%)**	1 (20.0%)	3 (33.3%)	10 (83.3%)	2 (50.0%)	**0.039**	–
**Duration of total ATB use (days)**	0.0 ± 17^a^	9.0 ± 9^ac^	10.0 ± 6^ab^	24.0 ± 17^b^	**0.011**	–
**Dexamethasone use, yes (%)**	0 (0.0%)	8 (88.9%)	12 (100.0%)	4 (100.0%)	**<0.001**	–
**Remdesivir use, yes (%)**	0 (0.0%)	6 (66.7%)	12 (100%)	3 (75.0%)	**<0.001**	–
**Pneumonia, yes (%)**	3 (60.0%)	9 (100.0%)	12 (100.0%)	4 (100.0%)	**0.037**	–
**C_t_ value, RdRp gene**	20.25 ± 14.18	27.53 ± 8.75	27.07 ± 6.82	21.55 ± 7.72	0.218	–
**Charlson comorbidity index**	4.0 ± 3.0	3.0 ± 3.0	2.5 ± 4.0	2.0 ± 5.0	0.541	0.0 ± 2.0
Underlying diseases						
Hypertension	3 (60.0%)	1 (11.1%)	5 (41.7%)	2 (50.0%)	0.234	–
Myocardial infarction	0 (0.0%)	1 (11.1%)	1 (8.3%)	0 (0.0%)	>0.999	–
Cerebrovascular attack	0 (0.0%)	0 (0.0%)	0 (0.0%)	1 (25.0%)	0.133	–
Dementia	1 (20.0%)	0 (0.0%)	0 (0.0%)	1 (25.0%)	0.083	–
Diabetes mellitus	4 (80.0%)	3 (33.3%)	3 (25.0%)	2 (50.0%)	0.214	–
Hemiplegia	0 (0.0%)	0 (0.0%)	0 (0.0%)	1 (25.0%)	0.133	–
End-stage renal disease	2 (40.0%)	0 (0.0%)	0 (0.0%)	0 (0.0%)	**0.037**	–

A, improvement from mild (maintain OS 4 from admission to discharge); B, improvement from moderate (improved from OS 5 to OS 4); C, improvement from severe (improved from OS 6 to OS 4); D, deterioration (aggregated from OS 4 or 5 to OS 7 or 8).

BMI, body mass index; COVID-19, coronavirus disease; ATB, antibiotics; C_t_, cycle threshold; RdRp gene, RNA-dependant RNA polymerase gene. Superscripts (^a,b^). For a particular variable, mode means with different superscript are significantly (p <0.05) different. Mode means with same superscripts are not significantly (p >0.05) different. When only one contrast is significant, one of the cells means has no superscript attached. The pair of cell means that is significant has different superscripts.

Cases with p-value <0.05 are indicated in bold.

### 3.2 Serial changes of gut microbiota, cytokines, and antibody

**Figure 1:**
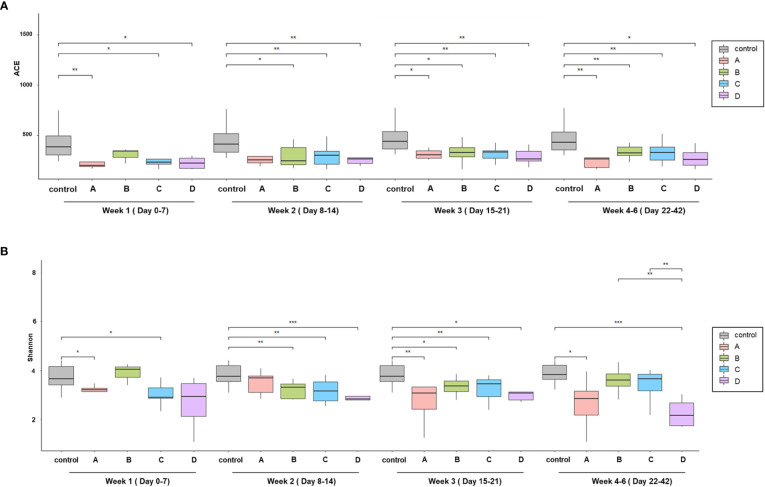
Comparison of alpha diversity among the prognosis groups. **(A)** Species richness by ACE index and **(B)** species diversity by the Shannon index. Boxplots show the change of alpha diversity serially between control, (A–D) groups. *p < 0.05; **p, 0.01–0.001; ***p < 0.001.

With regard to the alpha diversity (species richness calculated by the ACE index), a significant difference was observed between the control group and each COVID-19 group; however, the alpha diversity of group B in week 1 and group A in week 2 were indistinguishable from that of the control group (**Figure 1A**). Based on the Shannon–Wiener index, the control group, had the most diverse gut microbiota. In contrast, group D had the least diversity (**Figure 1B**). There were significant differences between the control group and each COVID-19 group until week 3. However, this difference was significantly alleviated at week 4, although a large difference in alpha diversity was maintained between group D and the control group. When analyzed using the ACE index, the difference in alpha diversity between the control group and each COVID-19 group persisted until week 4. In contrast to groups B and C, the alpha diversity of group A was significantly lower than that of the control group throughout the study period, despite their mild disease severity. This difference might be related to the underlying medical conditions: group A had a higher Charlson comorbidity index, which was negatively correlated with the alpha diversity (**Table 1** and **Supplementary Figure 1**).

**Figure 2:**
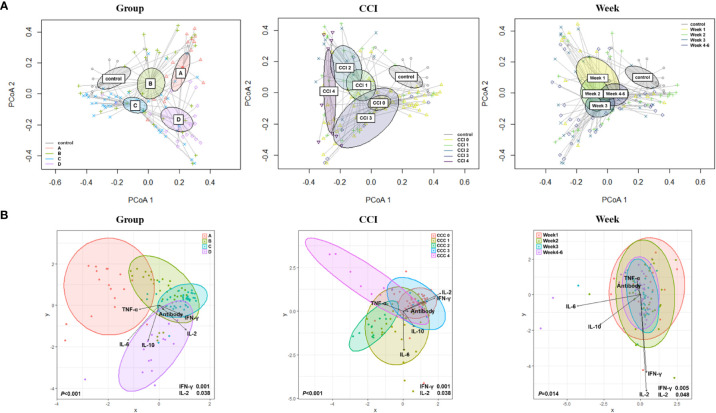
Beta diversity based on the clinical course, Charlson comorbidity index, and elapsed time (week). **(A)** Principal coordinate analysis (PCoA) using Bray–Curtis distances. **(B)** Canonical Correlation Analysis (CCA) with interferon (IFN)-γ; interleukin (IL)-2; IL-10; tumor necrosis factor-alpha (TNF-α); and anti-S immunoglobulin G (IgG) antibody.

For analyzing beta diversity by clinical course, principal coordinate analysis using Bray–Curtis distances was performed and revealed significant intergroup differences (*p*=0.003; **Figure 2A**). There was a significant difference in beta diversity according to the Charlson comorbidity index (*p*<0.001) and over time (weeks) after hospitalization (*p*<0.001). For a comprehensive analysis, we analyzed changes in the microbiome of the four groups over time after symptom onset (**Supplementary Figure 2**). During the early periods, the change in beta diversity was unremarkable within the first 7 days (week 1) after symptom onset (*p*=0.218). However, intergroup differences became apparent over time at week 2 (*p*=0.017). After the 15th day of symptom onset (weeks 3 and 4), the intergroup difference disappeared (*p*=0.116 and *p*=0.133 at weeks 3 and 4, respectively). The groups with clinical improvement (groups B and C) and the control group showed closer microbial composition at the convalescent stage, whereas in group D, whose clinical course worsened, microbial composition remained far from that of the control group. In group A, the intergroup distance was maintained far from that of the control group despite the clinical improvement, which might be due to the underlying medical conditions.

**Figure 3:**
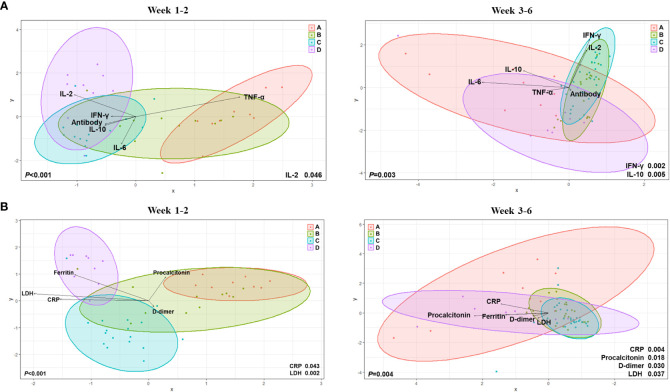
Canonical Correlation Analysis of intergroup differences at weeks 1 to 2 and weeks 3 to 6. **(A)** Canonical Correlation Analysis (CCA) with regard to the microbiome, cytokines, and anti-S immunoglobulin G (IgG) antibodies. **(B)** CCA for microbiome and inflammatory markers over time.

By using canonical correlation analysis (CCA), the gut microbiota composition of 30 patients with COVID-19 was visualized by prognostic groups with five serum cytokines – IFN-γ, IL-2, IL-10, TNF-α, and anti-SARS-CoV-2 S immunoglobulin G (IgG) titers (**Figure 2B**). Among these, two cytokines were significantly correlated with the microbiota composition in the clinical groups, as shown in **Figure 2B** (IFN-γ, *p*<0.001; IL-2, *p*=0.038). Furthermore, CCA showed a significant correlation between the gut microbiota and cytokines when analyzed using the Charlson comorbidity index (IFN-γ, *p*<0.001; IL-2, *p*=0.038) and the time that had elapsed after symptom onset (IFN-γ, *p*=0.005; IL-2, *p*=0.048) (**Figure 2B**). Additionally, we conducted analyses over time with regard to the microbiome, cytokines, and anti-S IgG antibodies (**Figure 3A**). The results showed that cytokine levels differed significantly between the groups at week 1-2 (IL-2, *p*=0.046) and week 3-6 (IFN-γ, *p*=0.002; IL-10, *p*=0.005), whereas anti-S IgG antibody titers were similar. Furthermore, the CCA was conducted for microbiome and inflammatory markers over time (**Figure 3B**); the increments in C-reactive protein (CRP) (*p*=0.043) and LDH (*p*=0.002) levels differed significantly between the groups at week 1-2, whereas CRP (*p*=0.004), LDH (*p*=0.037), procalcitonin (*p*=0.018), and D-dimer (*p*=0.030) levels significantly differed among the groups at week 3-6.

When all factors were integrated and analyzed weekly (**Supplementary Figure 3**), the ferritin levels increased significantly in group D at weeks 2 (*p*=0.014) and 3 (*p*=0.007). At week 4, the IFN-γ (*p*=0.022), IL-10 (*p*=0.010), and IL-6 (*p*=0.014) levels differed significantly between the prognosis groups. At the early stage of infection (week 1-2), IFN-γ and IL-6 levels were lower in groups C and D than in groups A and B (**Supplementary Table 3** and **Supplementary Figure 3**). In comparison, in the later stage (week 4-6), the IFN-γ and IL-6 levels decreased in groups A and B. In contrast, they increased in groups C and D. In the early stage, the IL-2 levels were low in all groups but became significantly higher in group D at week 4. TNF-α levels were lower in the early stage and higher in the late stage in group D than in the other groups. The IL-10 levels were higher in group D than in the other groups, particularly at week 4. The anti-S IgG antibody titer was significantly higher in group C than in the other groups at week 1, but became similar from week 2 (**Supplementary Table 4**).

### 3.3 Gut microbiota as a potential predictor of COVID-19 prognosis

Next, we assessed the relative abundance of the bacterial taxa to explore potential taxonomic markers for predicting COVID-19 prognosis. As group A included two people with ESRD as the underlying disease, microbiota whose ratio decreased from groups B to D and who showed a linear discriminant analysis (LDA) effect size >2.5 were considered good taxonomic markers (**Table 2**). Conversely, factors with an increasing ratio from groups B to D and an LDA effect size >2.5 were regarded as poor taxonomic markers.

**Table 2:** Good taxonomy prognostic markers in patients with COVID-19.

Taxon name	Taxon rank	Taxonomy	LDA effect size	*P* value
Peptoniphilus lacrimalis	Species	Bacteria: Firmicutes: Tissierellia: Tissierellales: Peptoniphilaceae: Peptoniphilus	4.00551	**0.02019**
Anaerococcus prevotii	Species	Bacteria: Firmicutes: Tissierellia: Tissierellales: Peptoniphilaceae: Anaerococcus	4.00885	**0.00736**
Dialister	Genus	Bacteria: Firmicutes: Negativicutes: Veillonellales: Veillonellaceae	3.97856	**0.00414**
JRNA_g	Genus	Bacteria: Firmicutes: Clostridia: Clostridiales: Mogibacterium_f	3.46909	**0.03179**
JRNA_s	Species	Bacteria: Firmicutes: Clostridia: Clostridiales: Mogibacterium_f: JRNA_g	3.44772	**0.03179**

LDA, linear discriminant analysis. Taxonomy prognostic markers commonly found at two early time points (0-7 days and 8-14 days) are presented. LDA effect size and p value are expressed as values of 0-7 days.

Cases with p-value <0.05 are indicated in bold.

We used LDA effect size to identify prognosis-related taxonomic markers during the early period (weeks 1 to 2) after symptom onset. We found that *Peptoniphilus lacrimalis* (LDA effect size: 4.00551, *p*=0.02019), *Anaerococcus prevotii* (LDA effect size: 4.00885, *p*=0.00736), genus *Dialister* (LDA effect size: 3.97856, *p*=0.00414), *Mogbacterium JRNA_g* (LDA effect size: 3.46909, *p*=0.03179), and *Mogibacterium JRNA_g JRNA_s* (LDA effect size: 3.44772, *p*=0.03179) were good prognosis-related taxonomic markers. However, there were no taxonomic markers that significantly indicated a poor prognosis.

### 3.4 Functional prediction of COVID-19 prognosis based on predominant taxa

We investigated functional biomarkers for predicting prognosis similarly to the process followed for taxonomic markers (**Table 3**). The Kyoto Encyclopedia of Genes and Genomes (KEGG) orthologs K07497 (putative transposase, LDA effect size: 3.488876, *p*=0.015) and K07483 (transposase, LDA effect size: 3.340157, *p*=0.015) were identified as good functional biomarkers.

**Table 3:** Functional prognostic markers in patients with COVID-19.

Good functional markers			
Ortholog	Definition	LDA effect size	*P* value
K07497	putative transposase	3.488876	**0.014721**
K07483	transposase	3.340157	**0.037935**
Poor functional markers			
Ortholog	Definition	LDA effect size	*P* value
K01223	6-phospho-beta-glucosidase	2.861785	**0.03848**
K21064	5-amino-6-(5-phospho-D-ribitylamino) uracil phosphatase	2.574397	**0.038698**
K02756	PTS system, beta-glucoside-specific IIB component	2.524706	**0.010420**
K03488	beta-glucoside operon transcriptional antiterminator	2.515448	**0.006972**
Module	Definition	LDA effect size	*P* value
M00271^c^	PTS system, beta-glucoside-specific II component	3.210534	**0.010931**
M00632^c^	Galactose degradation, Leloir pathway, galactose => alpha-D-glucose-1P	2.968915	**0.010707**
Pathway	Definition	LDA effect size	*P* value
ko00500^c^	Starch and sucrose metabolism	3.415623	**0.017574**
ko00052^d^	Galactose metabolism	3.393237	**0.042615**

LDA, linear discriminant analysis. Good functional prognostic markers commonly found at two early time points (0-7 days and 8-14 days) are presented, while poor functional prognostic markers were only found at a very early time point (0-7 days). LDA effect size and p value are expressed as values of 0-7 days.

Superscripts (^c,d^). For a particular variable, we used PICRUSt or MinPath algorithm. Therefore, mode means with the same superscripts are the results using the same algorithm.

Cases with p-value <0.05 are indicated in bold.

In contrast, the KEGG orthologs K01223 (6-phospho-β-glucosidase, LDA effect size: 2.861785, *p*=0.038), K21064 (5-amino-6-[5-phospho-D-ribitylamino] uracil phosphatase, LDA effect size: 2.574397, *p*=0.038), K02756 (phosphotransferase [PTS] system, beta-glucoside-specific IIB component, LDA effect size: 2.524706, *p*=0.010), and K03488 (beta-glucoside operon transcriptional anti-terminator, LDA effect size: 2.515448, *p*=0.007) were poor functional biomarkers. The PICRUSt algorithm revealed that the KEGG modules M00271 (PTS system, beta-glucoside-specific IIB component, LDA effect size: 3.210534, *p*=0.011), M00632 (galactose degradation, Leloir pathway, galactose ≥ alpha-D-glucose-1P, LDA effect size: 2.968915, *p*=0.011), and ko00500 (starch and sucrose metabolism, LDA effect size: 3.415623, *p*=0.018) were dominant in the poor prognosis group. Using the MinPath algorithm, the KEGG pathway ko00052 (galactose metabolism, LDA effect size: 3.393237, *p*=0.043) was abundantly detected in the poor prognosis group.

### 3.5 Correlation of prognosis-related taxonomic markers with dietary and nutritional factors

**Figure 4:**
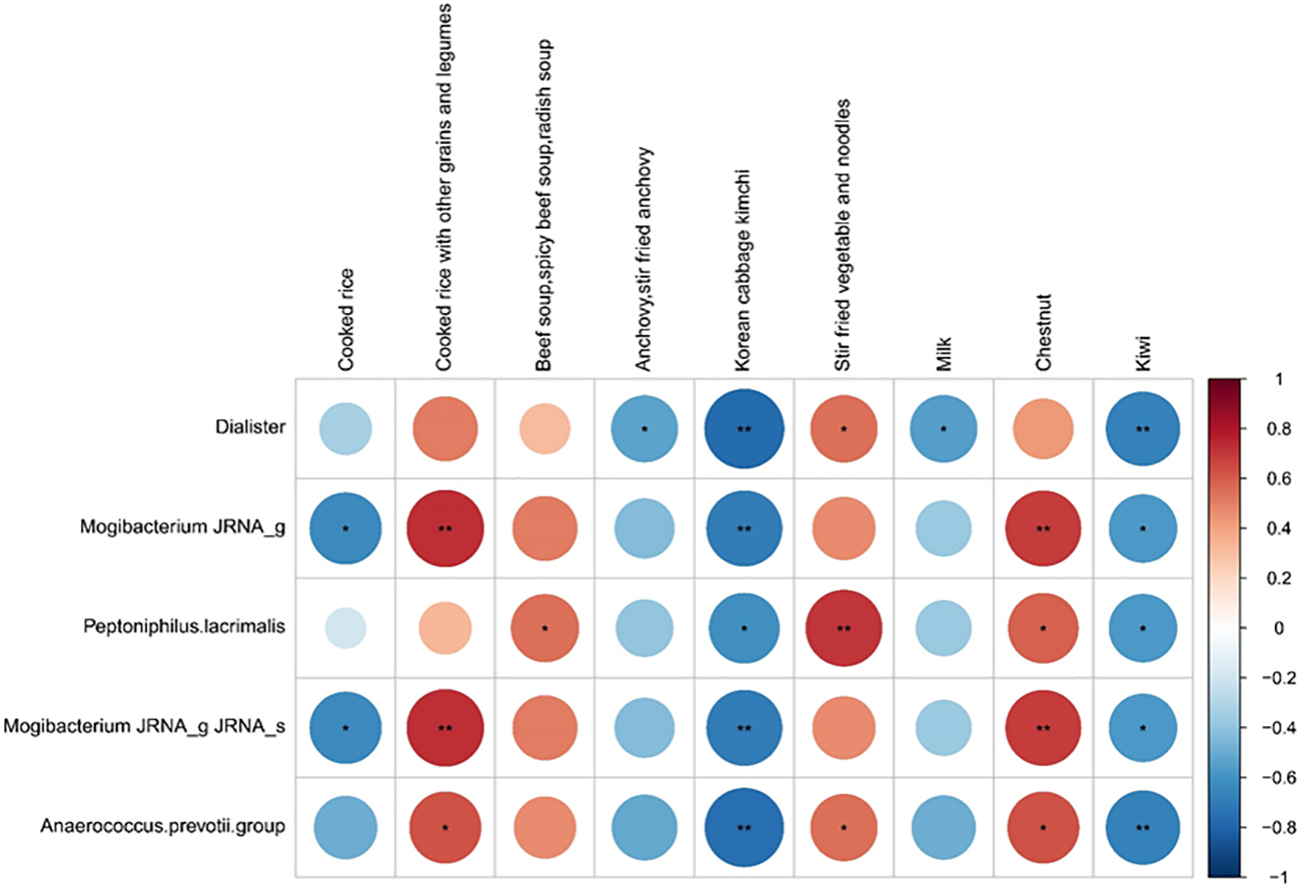
Correlation between good prognosis-related taxonomic biomarkers and nine listed food items. Spearman rank analysis was conducted to evaluate the association between good prognosis-related taxonomic biomarkers and food items. The color gradients indicate the degree of correlation from red (positive correlation) to blue (negative correlation). *p < 0.05; **p, 0.01–0.001.

We investigated the possible association between dietary components and COVID-19 prognosis. Correlation analysis was conducted between five good taxonomic markers and 112 food items (**Figure 4** and **Supplementary Figure 4**). Among the 112 food items, good taxonomic markers were negatively correlated with cooked rice, whereas they showed a positive correlation with cooked rice along with other grains and legumes. In addition, good taxonomic markers increased with the intake of beef soup, stir-fried vegetables, noodles, and chestnuts, whereas they decreased with the intake of anchovies, Korean cabbage kimchi, milk, and kiwi.

## 4 Discussion

In this study, we found that the gut microbiota–immune axis might play an important role in the prognosis of patients with COVID-19. The composition of the gut microbiota differed depending on the severity of COVID-19, and individual microbiota can affect immune, inflammatory, and metabolic responses to viruses. The genera Dialister, Peptoniphilus lacrimalis, and Anaerococcus prevotii are taxonomic markers that reflect a good prognosis. In patients with SARS-CoV-2 infection, high starch, sucrose, and galactose metabolism in the gut microbiota were associated with a poor prognosis.

Alpha diversity was lower in the SARS-CoV-2–infected patient groups than in the control group. When compared over time, the alpha diversity of the groups with a good prognosis (A–C) was gradually restored, but not to the level of healthy controls until weeks 4-6 post-infection. In comparison, decreased alpha diversity persisted in the poor prognostic group (D). As ACE2, the main receptor of SARS-CoV-2, is highly expressed as a neutral amino acid transporter in the gastrointestinal tract; the virus can invade the gut, disrupting mucosal integrity and microbial dysbiosis (10, 11). According to a previous meta-analysis, SARS-CoV-2 RNA was detectable in the feces for a mean duration of 21.8 days after the diagnosis of COVID-19, which was 7 days longer than the detectability in the respiratory specimens (12). When SARS-CoV-2 invades the gastrointestinal tract, ACE2 expression decreases, resulting in gut microbiota dysbiosis (3). In patients with COVID-19, the severity of gut microbiota dysbiosis is likely to be in accordance with disease severity. Thus, microbial dysbiosis might contribute to the aggravation of COVID-19 while simultaneously reflecting a poor prognosis.

Based on clinical findings, response to therapy, and clinical outcomes, COVID-19 can be subdivided into three phases: viral, inflammatory, and recovery (13, 14). Thus, an appropriate cytokine response during the viral phase and immune modulation for the inflammatory phase would be crucial for the prognosis of COVID-19. We measured and analyzed serial changes in the levels of the five cytokines according to COVID-19 progression. In the initial stage of infection, groups C and D had lower levels of the proinflammatory cytokines IL-6, TNF-α, and IFN-γ and higher levels of anti-inflammatory cytokine IL-10 than the other groups. After the viral invasion, the innate immune response directs infected cells to secrete several proinflammatory cytokines, such as IL-6, TNF-α, and IFN-γ (15). Therefore, proper secretion of proinflammatory cytokines in the early stage is crucial for eliminating the invading virus. We suggest that insufficient early release of proinflammatory cytokines may influence the prognosis of COVID-19. A later increase in the secretion of proinflammatory cytokines, which was initially low, reflected the hyperinflammatory response during the inflammatory phase (2 weeks after symptom onset). When we compared the weekly changes in microbiota, cytokine, and antibody levels according to the prognosis group, the IFN-γ, IL-6, and IL-10 levels markedly differed among the four prognostic groups. These results suggest that hyperinflammation in the late phase of COVID-19 may be associated with poor prognosis.

In this study, serial changes in the gut microbiota composition were related to cytokine responses according to the clinical course of COVID-19. These correlations may be supported by the characteristics of the taxonomic markers that reflected the prognosis of COVID-19 in this study. Propionate, a major short-chain fatty acid, is mainly produced through the succinate decarboxylation pathway that involves the Dialister species (16). Propionate downregulates several inflammatory cytokines and chemokines, such as TNF-α (17). The high abundance of the genus Dialister correlated with reduced serum levels of the proinflammatory cytokine IL-6, particularly in association with the consumption of whole grains (18–20). In addition, Zhang et al. found that the genus Dialister was negatively correlated with IL-2 and IL-6 levels in kid goats (21). Peptoniphilus lacrimalis and Anaerococcus prevotii are indole-producing species (22). Several studies have demonstrated that indole and its tryptophan derivatives regulate epithelial integrity and modulate the immune response by binding to the aromatic hydrocarbon receptor on epithelial and immune cells (23–27). The roles of Mogibacterium JRNA_g and Mogibacterium JRNA_g JRNA_s should be investigated further.

With regard to the functional biomarkers, we found that the starch, sucrose, and galactose metabolism pathways were highly activated in the poor prognosis group. Our findings show that obesity and diabetes mellitus are risk factors for severe COVID-19 (28, 29). Viruses, including SARS-CoV-2, alter host metabolism to optimize host conditions for effective replication and spread. In a large cohort study, uncontrolled blood glucose levels correlated with poor prognosis (28). The increased glucose production may enhance SARS-CoV-2 entry and subsequent replication (30). Codo et al. explored the dose-dependent increase in glucose concentrations that potentiate SARS-CoV-2 replication in the SARS-CoV-2-induced monocyte response. Moreover, the authors showed that elevated glucose levels directly promote SARS-CoV-2 replication, cytokine production in monocytes, and subsequent T-cell impairment (31). Our prognosis-related taxonomic markers support these results. Propionate, generated by the Dialister species-involved pathway, plays a significant role in glucose metabolism by promoting insulin secretion, improving insulin sensitivity, and activating intestinal gluconeogenesis (32–34). Peptoniphilus lacrimalis and Anaerococcus prevotiisms may promote insulin secretion and inhibit glucagon secretion through indole-mediated intestinal L-cell stimulation (35). Taken together, the gut microbiota may affect COVID-19 prognosis through metabolic pathways.

Dietary carbohydrate restriction improves glycaemic control, thereby mitigating COVID-19 (36, 37). We found that whole grain (cooked rice with other grains and legumes) intake increased good prognosis-related taxonomic markers, whereas intake of refined grain (cooked rice) and simple carbohydrates (milk and kiwi) decreased those markers. Diet could contribute to the prognosis of COVID-19 because the most significant factor that determines blood glucose levels is the consumption of dietary carbohydrates.

There are some limitations in this study. First, there was a remarkable difference in the ages between healthy controls and COVID patients. Second, the sample size of healthy controls was small. Third, both Alpha and Delta variant viruses circulated during the study periods; Alpha variant was dominant between January and May of 2021, and was gradually replaced by the Delta variant since June. Viral factor might have affected overblown variations in some results (38). Finally, long-term follow-up data was not available in cured patients.

In conclusion, this study showed that the gut microbiota and cytokines are closely related. Gut microbiota may influence immune responses and glucose metabolism, which in turn are involved in the prognosis of COVID-19. High starch, sucrose, and galactose metabolism in the gut microbiota during SARS-CoV-2 infection may increase glucose levels, thereby promoting SARS-CoV-2 replication and proinflammatory cytokine production. The gut microbiota is strongly implicated in host immune responses to SARS-CoV-2 and regulates timely cytokine activation. Early-inadequate and late-dysregulated cytokine responses are associated with poor prognosis of COVID-19.

## Data availability statement

The raw data supporting the conclusions of this article will be made available by the authors, without undue reservation. The datasets generated and/or analyzed during the current study are available in the NCBI repository, BioProject gut microbiome [Accession No. PRJNA941155].

## Ethics statement

The studies involving human participants were reviewed and approved by Institutional Review Board of Korea University Guro Hospital (2020GR0570) Institutional Review Board of Chungbuk National University Hospital (2020-12-021). The patients/participants provided their written informed consent to participate in this study.

## Author contributions

Conceptualization, HS, JK, JN, HC, WK, and JS. Methodology, HS, JK, Y-HH, EN, HSS, and SL. Formal analysis, HS, Y-HH, and SL. Investigation, HS, JK, HH, and JY. Data curation, HS and SL. Writing - original draft, HS, Y-HH, SL, and JS. Writing–review and editing, HS and JS. Visualization, HS and SL. Supervision, HS, SL, and JS. Funding acquisition, HS, SL, and JS. All authors contributed to the article and approved the submitted version.
