# Supplementary material for: Clinical implications of gut microbiota and cytokine responses in coronavirus disease prognosis
Source: Front Immunol. 2023 Mar 24;14:1079277. doi: 10.3389/fimmu.2023.1079277 (PMC10083496; doi:10.3389/fimmu.2023.1079277)
Supplement: Supplementary file 1 [file DataSheet_1.docx]

Supplementary Material

# Supplementary Method

**Microbiological analysis**

**DNA extraction, PCR amplification, and Sequencing for 16s rRNA**

Total DNA was extracted using the PowerFecal® Pro DNA kit (QIAGEN, GERMANY), following the manufacturer’s instructions. PCR amplification was performed using fusion primers targeting the V3–V4 regions of the 16S rRNA gene, with the extracted DNA used as the template. For bacterial amplification, the following fusion primers were used: 341F (5ʹ-AATGATACGGCGACCACCGAGATCTACAC-XXXXXXXXTCGTCGGCAGCGTC-AGATGTGTATAAGAGACAG-CCTACGGGNGGCWGCAG-3ʹ; the underlined sequence indicates the target region primer) and 805R (5ʹ- CAAGCAGAAGACGGCATACGAGAT-XXXXXXXXGTCTCGTGGGCTCGG-AGATGTGTATAAGAGACAG-GACTACHVGGGTATCTAATCC-3ʹ). The fusion primers were constructed in the following order: P5 (P7) graft binding, i5 (i7) index, Nextera consensus, sequencing adaptor, and target region sequence. PCR amplifications were carried out under the following conditions: Initial denaturation at 95 °C for 3 min, followed by 25 cycles of denaturation at 95 °C for 30 s, primer annealing at 55 °C for 30 s, and extension at 72 °C for 30 s, with a final elongation step at 72 °C for 5 min. The PCR products were confirmed by electrophoresis using 1% agarose gel followed by visualization using a Gel Doc system (Bio-Rad, Hercules, CA, USA). The amplified products were purified using the CleanPCR kit (CleanNA). Equal concentrations of the purified products were pooled, and short fragments (non-target products) were removed using the CleanPCR kit (CleanNA). The quality and product size were assessed on a Bioanalyzer 2100 system (Agilent, Palo Alto, CA, USA) using a DNA 7500 chip. Mixed amplicons were pooled, and the sequencing was performed at ChunLab, Inc. (Seoul, Korea), using the Illumina MiSeq Sequencing System (Illumina, San Diego, CA, USA), according to the manufacturer’s instructions.

**DNA analysis pipeline**

The raw reads were first quality-checked, and low quality (<Q25) reads were filtered using Trimmomatic ver. 0.32. After QC processing, paired-end sequence data were merged using the fastq_mergepairs command of VSEARCH ver.2.13.4. with default parameters. Next, the primers were trimmed using the Myers–Miller alignment algorithm (1) at a similarity cut-off of 0.8. Non-specific amplicons, i.e., those that did not encode 16S rRNA, were detected using the nhmmer algorithm (2) in the HMMER software package ver.3.2.1 with hmm profiles. Unique reads were extracted, and redundant reads were clustered with the unique reads using the deep-full-length command of VSEARCH (3). The EzBioCloud 16S rRNA database (4) was used for taxonomic assignment using the usearch_global command of VSEARCH (3), followed by a more precise pairwise alignment (1). Chimeric reads were filtered to obtain reads with <97% similarity by reference-based chimeric read detection using the UCHIME algorithm (5) and the non-chimeric 16S rRNA database from EzBioCloud. After chimeric filtering, reads that were not identified to the species level (with <97% similarity) in the EzBioCloud database were compiled, and the cluster_fast command (3) was used to perform de novo clustering to generate additional operational taxonomic units (OTUs). Next, OTUs with single reads (singletons) were omitted from further analysis. The secondary analyses, including diversity calculation and biomarker discovery, were performed using in-house programs of ChunLab, Inc. The alpha diversity indices (ACE (6), Chao1 (7), Jackknife (8), Shannon (9), NPShannon (10), Simpson (9), and Phylogenetic diversity (11)) were estimated. To visualize the sample differences, beta diversity distances were calculated using several algorithms (Jensen–Shannon (12), Bray–Curtis (13), Generalized UniFrac (14), and Fast UniFrac (15)). Functional profiles were predicted using the PICRUSt (16) and MinPath (17) algorithms and taxonomic and functional biomarkers were discovered using statistical comparison algorithms (linear discriminant analysis effect size [LEfSe])(18). All microbiome count data were normalized relative to 18,000 read counts before further use. All the aforementioned analyses were performed using the EzBioCloud 16S-based MTP, which is ChunLab’s bioinformatics cloud platform.

# Supplementary Figures and Tables

## Supplementary Figures


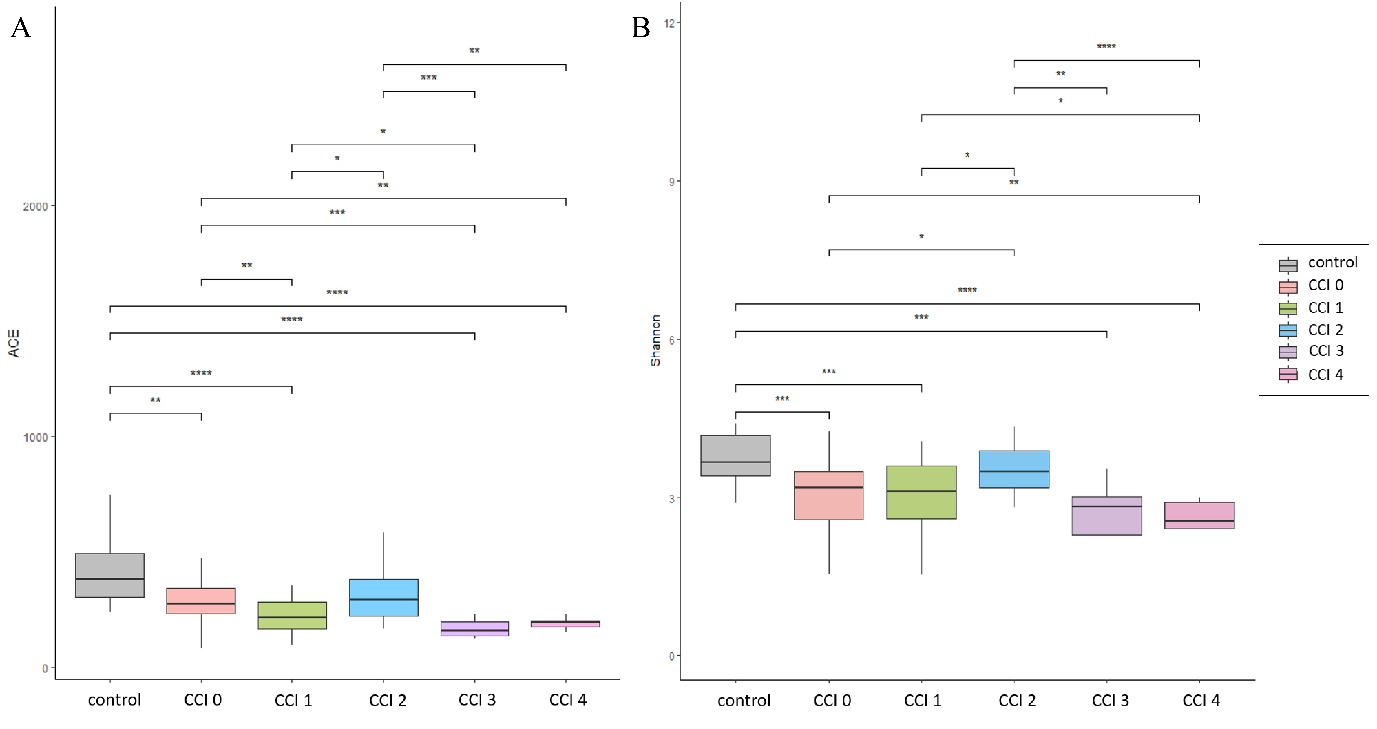


**Supplementary Figure 1.** Comparison of alpha diversity by the Charlson comorbidity index (CCI). (A) species richness by ACE and (B) species diversity by the Shannon Index. Boxplots shows the alpha diversity in healthy controls and SARS-CoV-2–infected patients, stratified by the CCI (CCI0, CCI1, CCI2, CCI3 and CCI4). *p<0.05; **p, 0.01–0.001; ***p<0.001.

**
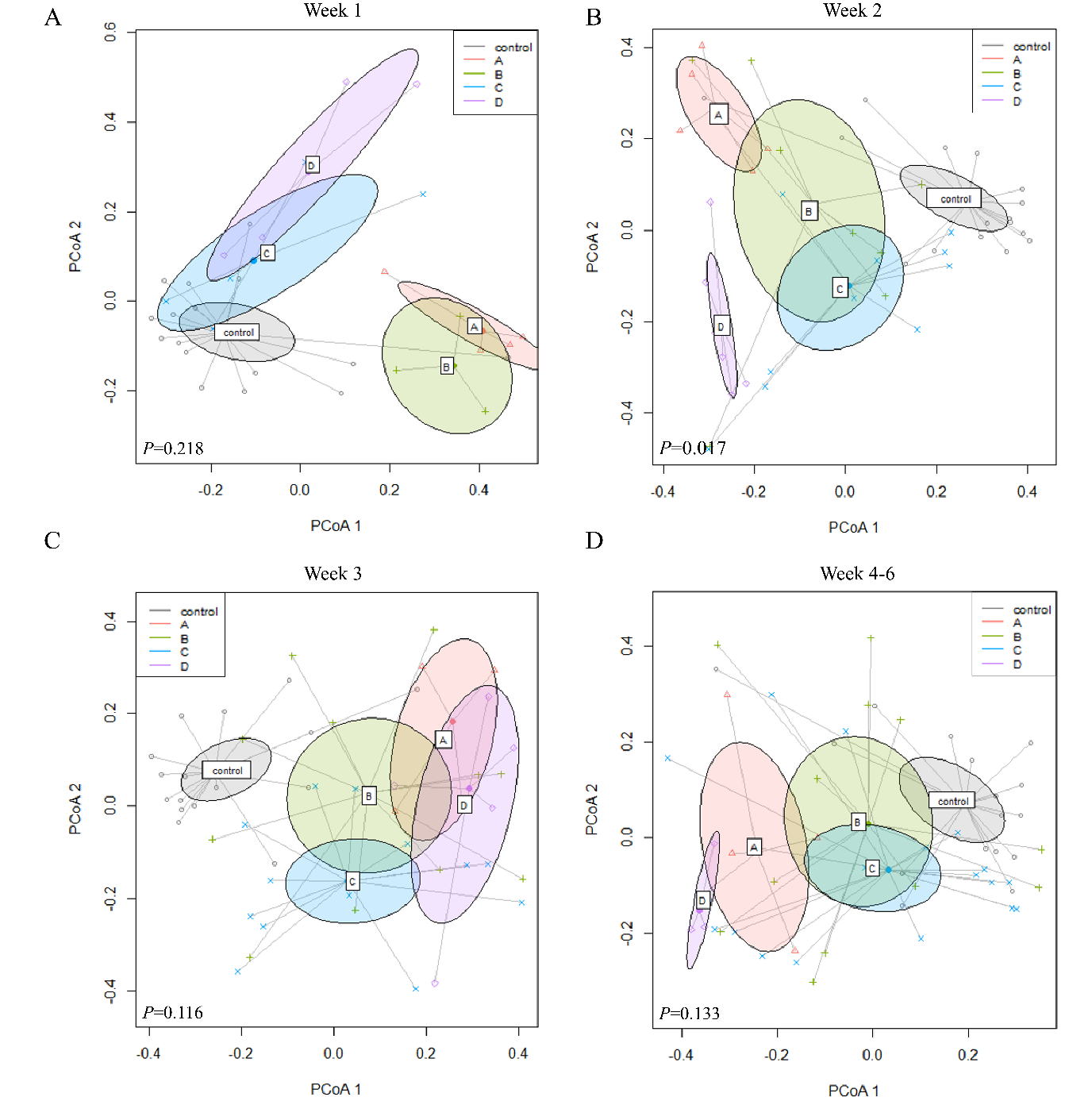
**

**Supplementary Figure 2.** Comparison of beta diversity per week between healthy controls and the prognosis groups. Principal coordinate analysis (PCoA) using Bray–Curtis distances at Week 1 (A), Week 2 (B), Week 3 (C), and week 4–6 (D).

**
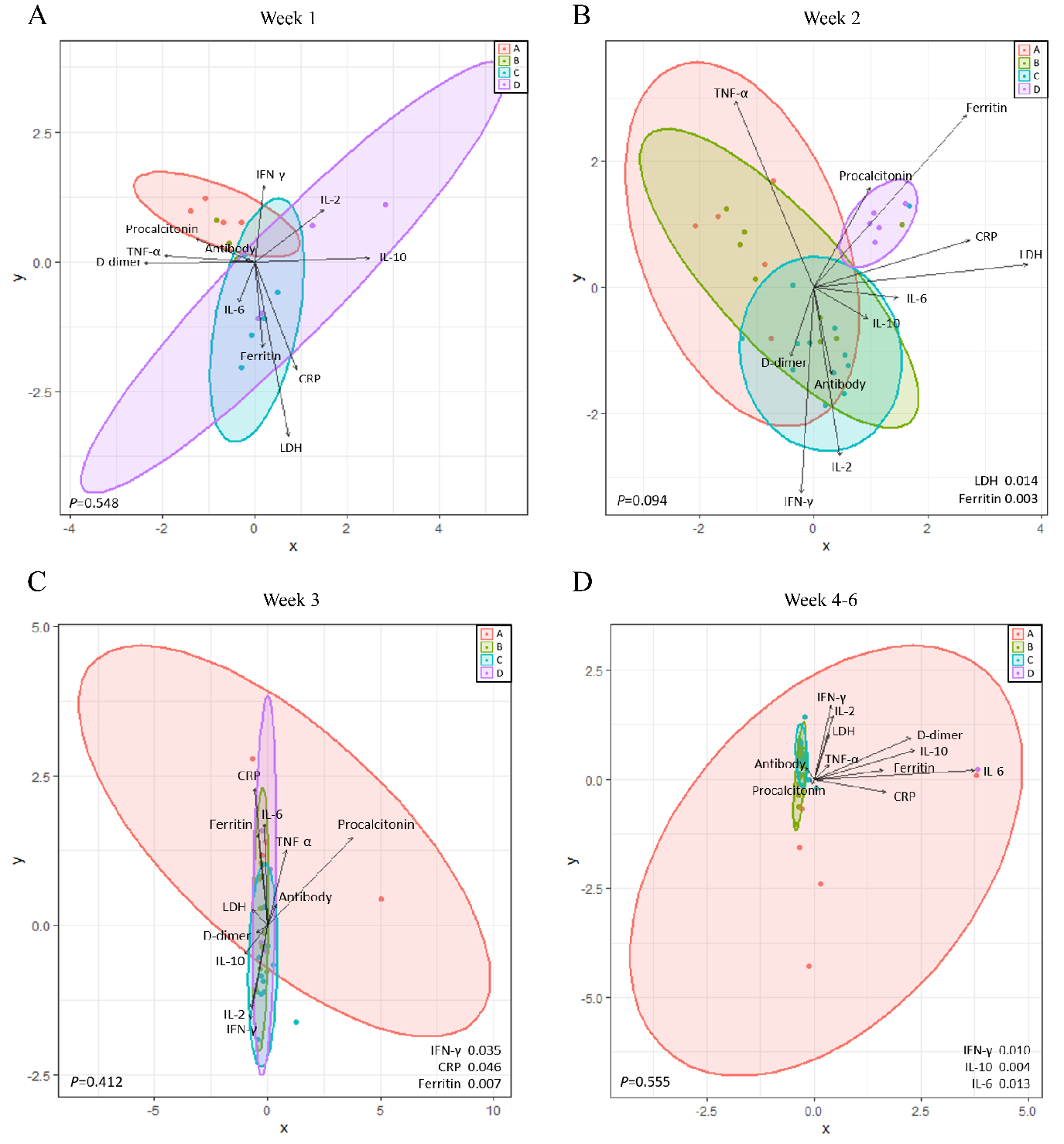
**

**Supplementary Figure 3.** Canonical Correlation Analysis of prognosis group-based gut microbiome with cytokines, inflammatory markers, and anti-S IgG antibodies at Week 1 (A), Week 2 (B), Week 3 (C), and week 4–6 (D).

**
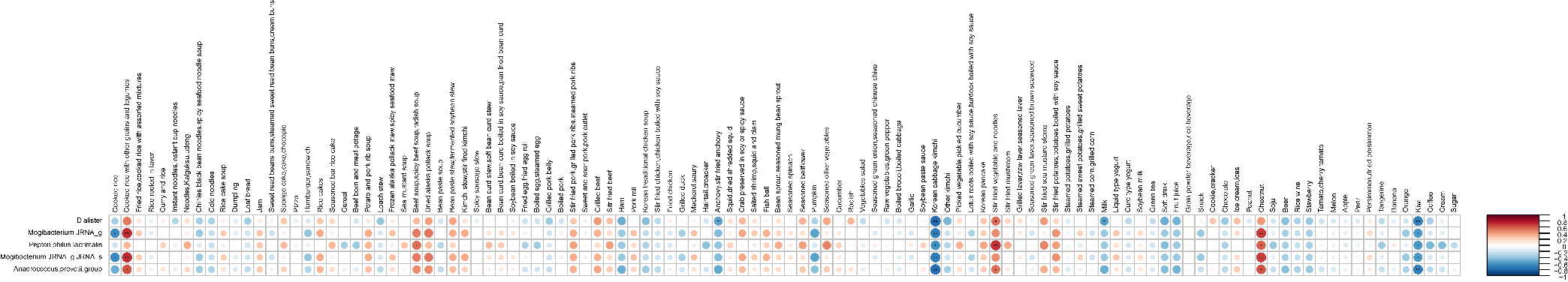
**

**Supplementary Figure 4.** Correlation between good prognosis-related taxonomic biomarkers and 112 listed food items. Spearman rank analysis was conducted to evaluate the association between good prognosis-related taxonomic biomarkers and food items. The color gradients indicate the degree of correlation from red (positive correlation) to blue (negative correlation). *p<0.05; **p, 0.01–0.001; ***p<0.001.

## Supplementary Tables

| **Table S1. National Institute of Allergy and Infectious Disease Ordinal Scale (NIAID-OS)** | | |
| --- | --- | --- |
| **Patients** | **NIAID-OS** | **Description** |
|  | **1** | Not hospitalized, no limitations on activities |
|  | **2** | Not hospitalized, no limitations on activities and/or requiring home oxygen |
|  | **3** | Hospitalized, not requiring supplemental oxygen- no longer requires ongoing medical care |
| Population enrolled | **4** | Hospitalized, nor requiring supplemental oxygen- requiring ongoing medical care |
|  | **5** | Hospitalized, requiring supplemental oxygen |
|  | **6** | Hospitalized, on non-invasive ventilation or high flow oxygen devices |
|  | **7** | Hospitalized, on mechanical ventilation or ECMO |
|  | **8** | Death |
| ECMO, extracorporeal membrane oxygenation. | | |

| **Table S2. Comparison of laboratory test results of subjects** | | | | | | |
| --- | --- | --- | --- | --- | --- | --- |
| **Characteristics** | **COVID-19 cases (*n* = 30)** | | | | ***P* value** | **Healthy control**  **(*n* = 15)** |
|  | **A (*n* = 5)** | **B (*n* = 9)** | **C (*n* = 12)** | **D (*n* = 4)** |  |  |
| WBC (10^3^cells/uL) | 4.08±8.63 | 6.20±4.43 | 7.65±3.83 | 6.95±4.95 | 0.666 | 6.00±2.00 |
| Neutrophil (cells/uL) | 2212.50±6674.75 | 4622.02±3596.52 | 5580.25±4341.00 | 6002.80±4475.68 | 0.711 | 3405.70±2185.58 |
| Lymphocyte (cells/uL) | 888.54±1850.38 | 456.57±636.71 | 694.35±719.55 | 336.50±360.43 | 0.083 | 1867.20±991.00 |
| Monocyte (cells/uL) | 452.88±636.28 | 462.80±445.96 | 486.50±573.75 | 266.50±485.37 | 0.489 | 408.80±181.60 |
| Hemoglobin (g/dL) | 12.7±1.9 | 13.7±2.0 | 12.3±2.4 | 13.1±5.0 | 0.557 | 14.0±2.0 |
| MCV (fL) | 94.5±10.1 | 90.6±3.2 | 91.4±5.0 | 94.1±5.1 | 0.283 | 90.7±7.2 |
| MCH (pg) | 31.7±2.6 | 30.2±1.6 | 31.1±1.8 | 31.7±2.9 | 0.410 | 30.3±2.6 |
| MCHC (g/dL) | 32.6±1.1 | 33.4±0.9 | 33.8±1.1 | 33.5±1.5 | 0.075 | 32.95±1.3 |
| Platelet count (10^3^/uL) | 152.0±131.0 | 177.0±122.0 | 189.5±91.0 | 165.0±75.0 | 0.374 | 282.0±90.0 |
| BUN (mg/dL) | 46.4±40.8 | 14.6±9.2 | 16.4±4.3 | 17.5±10.7 | 0.206 | 16.1±6.5 |
| Creatinine (mg/dL) | 1.12±7.53 | 0.69±0.22 | 0.67±0.39 | 0.62±0.26 | 0.174 | 0.67±0.28 |
| Total protein (g/dL) | 6.3±0.8 | 6.4±1.3 | 6.2±0.7 | 6.7±1.6 | 0.822 | N/A |
| Albumin (g/dL) | 3.5±1.1 | 3.5±0.6 | 3.4±0.5 | 3.4±0.7 | 0.519 | 4.3±0.3 |
| HDL cholesterol (mg/dL) | 50.0±25.0 | 36.0±24.0 | 41.5±19.0 | 44.5±24.0 | 0.703 | 53.5±17.0 |
| LDL cholesterol (mg/dL) | 73.0±74.0 | 67.0±34.0 | 65.5±36.0 | 101.0±91.0 | 0.412 | 129.0±45.0 |
| Triglyceride (mg/dL) | 110.0±150.0 | 77.0±75.0 | 87.5±48.0 | 146.0±135.0 | 0.726 | 74.5±93.0 |
| AST (IU/L) | 20.0±40.0 | 43.0±21.0 | 49.5±30.0 | 38.0±15.0 | 0.164 | 24.5±13.0 |
| ALT (IU/L) | 15.0±18.0 | 33.0±28.0 | 43.5±40.0 | 30.0±41.0 | 0.100 | 25.0±31.0 |
| Total bilirubin (mg/dL) | 0.30±0.16^a^ | 0.55±0.13^a^ | 0.51±0.27^a^ | 0.39±0.29^a^ | 0.039 | N/A |
| ESR (mm/hr) | 44.0±44.0 | 57.0±36.0 | 55.0±32.0 | 42.0±53.0 | 0.840 | N/A |
| CRP (mg/L) | 10.42±43.03 | 15.61±127.24 | 34.96±83.63 | 46.69±56.72 | 0.141 | 0.54±1.22 |
| Procalcitonin (ng/mL) | 0.27±0.63 | 0.08±0.12 | 0.065±0.05 | 0.09±0.09 | 0.286 | N/A |
| LDH (IU/L) | 525.0±225.0^a^ | 695.0±551.0^ab^ | 929.0±248.0^b^ | 818.5±441.0^ab^ | 0.033 | N/A |
| Ferritin (ng/mL) | 418.30±430.03 | 562.70±389.73 | 592.35±607.00 | 1236.50±1980.15 | 0.895 | N/A |
| A, recovered from mild COVID-19; B, improved from moderate to mild severity; C, improved from severe to mild severity; D, deterioration.  WBC, white blood cell; ANC, absolute neutrophil count; MCV, mean corpuscular volume; MCH, mean corpuscular hemoglobin; MCHC, mean corpuscular hemoglobin concentration; BUN, blood urea nitrogen; HDL, high density lipoprotein; LDL, low density lipoprotein; AST, aspartate transaminase; ALT, alanine transaminase; GGT, gamma-glutamyl transferase; CRP, C-reactive protein; LDH, lactate dehydrogenase; N/A, not available.  *Superscripts* (^a,b^). For a particular variable, mode means with different superscript are significantly (*p* <0.05) different. Mode means with same superscripts are not significantly (*p* >0.05) different. When only one contrast is significant, one of the cells means has no superscript attached. The pair of cell means that is significant has different superscripts.  Continuous variables are shown as median ± interquartile range (IQR) and categorical variables as numbers (percentage). | | | | | | |

| **Table S3. Changes in cytokine levels according to time elapsed after symptom onset in patients with COVID-19** | | | | | | | |
| --- | --- | --- | --- | --- | --- | --- | --- |
| **Week 1** | | | | | | | |
| **Cytokine (pg/mL)** | **A** | **B** | **C** | **D** | ***P* value** |  |  |
| IFN-γ | 1.77±9.60 | 1.54±(N.A) | 0.65±10.82 | 0.00±11.13 | 0.945 |  |  |
| IL-10 | 1.44±2.70 | 4.24±(N.A) | 1.23±1.55 | 6.60±4.66 | 0.059 |  |  |
| IL-2 | 0.00±0.00 | 0.00±0.00 | 0.00±2.90 | 0.00±6.72 | 0.427 |  |  |
| IL-6 | 11.89±12.79 | 11.50±(N.A) | 2.59±6.30 | 9.40±64.35 | 0.116 |  |  |
| TNF-α | 8.21±11.94 | 2.95±(N.A) | 3.27±12.43 | 1.84±2.40 | 0.127 |  |  |
| **Week 2** | | | | | |  |  |
| **Cytokine (pg/mL)** | **A** | **B** | **C** | **D** | ***P* value** |  |  |
| IFN-γ | 1.55±5.96 | 0.00±0.32 | 3.67±11.62 | 0.00±5.01 | 0.230 |  |  |
| IL-10 | 0.00±7.06 | 0.64±1.87 | 0.65±2.18 | 3.21±5.15 | 0.355 |  |  |
| IL-2 | 0.00±0.00 | 0.00±0.00 | 1.18±5.47 | 0.00±4.65 | 0.079 |  |  |
| IL-6 | 3.63±20.25 | 3.01±14.60 | 5.98±18.78 | 16.96±34.24 | 0.434 |  |  |
| TNF-α | 6.49±10.63 | 3.46±1.02 | 2.32±3.43 | 1.95±4.52 | 0.098 |  |  |
| **Week 3** | | | | | |  |  |
| **Cytokine (pg/mL)** | **A** | **B** | **C** | **D** | ***P* value** |  |  |
| IFN-γ | 0.22±2.11 | 0.00±5.41 | 6.91±10.44 | 0.00±5.87 | 0.652 |  |  |
| IL-10 | 0.00±0.00^a^ | 0.00±1.95^ab^ | 1.07±1.53^ab^ | 1.61±2.20^b^ | 0.033 |  |  |
| IL-2 | 0.00±0.00 | 0.00±3.74 | 3.74±3.76 | 0.00±3.77 | 0.185 |  |  |
| IL-6 | 3.49±22.11 | 2.38±3.96 | 1.95±2.79 | 5.95±10.38 | 0.352 |  |  |
| TNF-α | 7.99±8.12 | 3.01±3.14 | 1.95±2.46 | 2.68±4.79 | 0.070 |  |  |
| **Week 4** | | | | | |  |  |
| **Cytokine (pg/mL)** | **A** | **B** | **C** | **D** | ***P* value** |  |  |
| IFN-γ | 0.00±2.69 | 0.43±8.69 | 6.91±9.42 | 11.62±(N.A) | 0.136 |  |  |
| IL-10 | 0.00±0.00^a^ | 0.00±0.64^a^ | 0.00±1.30^ab^ | 2.76±(N.A)^b^ | 0.011 |  |  |
| IL-2 | 0.00±0.00^a^ | 0.00±0.00^a^ | 0.33±5.47^a^ | 5.47±(N.A)^a^ | 0.027 |  |  |
| IL-6 | 3.44±41.84 | 1.76±1.60 | 1.46±2.10 | 10.32±(N.A) | 0.093 |  |  |
| TNF-α | 5.06±5.33 | 3.72±9.68 | 2.67±2.11 | 4.60±(N.A) | 0.058 |  |  |
| A, recovered from mild COVID-19; B, improved from moderate to mild severity; C, improved from severe to mild severity; D, deterioration.  IL, interleukin; TNF, tumor necrosis factor; IFN, interferon; N.A, not applicapable. *Superscripts* (^a,b^). For a particular variable, mode means with different superscript are significantly (*p* <0.05) different. Mode means with same superscripts are not significantly (*p* >0.05) different. When only one contrast is significant, one of the cells means has no superscript attached. The pair of cell means that is significant has different superscripts. Variables are shown as median ± interquartile range. | | | | | |  |  |

| **Table S4. Antibody titers changes by week from symptom onset in patients with COVID-19** | | | | | | | |
| --- | --- | --- | --- | --- | --- | --- | --- |
| **Anti-SARS-CoV-2 S IgG (U/mL)** | **A** | **B** | **C** | **D** | ***P* value** |  |  |
| **Week 1** | 0.4±0.0^a^ | 0.4±(N.A)^ab^ | 1.96±115.43^b^ | 0.4±0.10^ab^ | 0.024 |  |  |
| **Week 2** | 24.8±114.05 | 3.71±207.88 | 23.7±228.28 | 53.80±71.75 | 0.732 |  |  |
| **Week 3** | 160.55±959.97 | 153.0±338.30 | 236.0±484.0 | 328.0±324.70 | 0.696 |  |  |
| **Week 4** | 250.0±866.2 | 279.50±396.80 | 728.0±1212.0 | 728.0±(N.A) | 0.230 |  |  |
| A, recovered from mild COVID-19; B, improved from moderate to mild severity; C, improved from severe to mild severity; D, deterioration.  N.A, not applicapable. *Superscripts* (^a,b^). For a particular variable, mode means with different superscript are significantly (*p* <0.05) different. Mode means with same superscripts are not significantly (*p* >0.05) different. When only one contrast is significant, one of the cells means has no superscript attached. The pair of cell means that is significant has different superscripts. Variables are shown as median ± interquartile range. | | | | | |  |  |

## Supplementary References

1. Myers EW, Miller W. Optimal alignments in linear space. *Comput Appl Biosci.* (1988) 4:11-7. doi:10.1093/bioinformatics/4.1.11

2. Wheeler TJ, Eddy SR. nhmmer: DNA homology search with profile HMMs. *Bioinformatics.* (2013) 29:2487-9. doi:10.1093/bioinformatics/btt403

3. Rognes T, Flouri T, Nichols B, Quince C, Mahé F. VSEARCH: a versatile open source tool for metagenomics. *PeerJ.* (2016) 4:e2584. doi:10.7717/peerj.2584

4. Yoon SH, Ha SM, Kwon S, Lim J, Kim Y, Seo H, et al. Introducing EzBioCloud: a taxonomically united database of 16S rRNA gene sequences and whole-genome assemblies. *Int J Syst Evol Microbiol.* (2017) 67:1613-7. doi:10.1099/ijsem.0.001755

5. Edgar RC, Haas BJ, Clemente JC, Quince C, Knight R. UCHIME improves sensitivity and speed of chimera detection. *Bioinformatics.* (2011) 27:2194-200. doi:10.1093/bioinformatics/btr381

6. Chao A, Lee S-M. Estimating the Number of Classes via Sample Coverage. *Journal of the American Statistical Association.* (1992) 87:210-7. doi:10.1080/01621459.1992.10475194

7. Chao A. Estimating the population size for capture-recapture data with unequal catchability. *Biometrics.* (1987) 43:783-91

8. Burnham KP, Overton WS. Robust Estimation of Population Size When Capture Probabilities Vary Among Animals. *Ecology.* (1979) 60:927-36. doi:10.2307/1936861

9. Magurran AE. *Measuring biological diversity*: John Wiley & Sons; 2013.

10. Chao A, Shen T-J. Nonparametric estimation of Shannon’s index of diversity when there are unseen species in sample. *Environmental and ecological statistics.* (2003) 10:429-43

11. Faith DP. Conservation evaluation and phylogenetic diversity. *Biological conservation.* (1992) 61:1-10

12. Lin J. Divergence measures based on the Shannon entropy. *IEEE Transactions on Information theory.* (1991) 37:145-51

13. Beals EW. Bray-Curtis ordination: an effective strategy for analysis of multivariate ecological data. *Advances in ecological research.* (1984) 14:1-55

14. Chen J, Bittinger K, Charlson ES, Hoffmann C, Lewis J, Wu GD, et al. Associating microbiome composition with environmental covariates using generalized UniFrac distances. *Bioinformatics.* (2012) 28:2106-13

15. Hamady M, Lozupone C, Knight R. Fast UniFrac: facilitating high-throughput phylogenetic analyses of microbial communities including analysis of pyrosequencing and PhyloChip data. *The ISME journal.* (2010) 4:17-27

16. Ye Y, Doak TG. A parsimony approach to biological pathway reconstruction/inference for genomes and metagenomes. *PLoS computational biology.* (2009) 5:e1000465

17. Langille MG, Zaneveld J, Caporaso JG, McDonald D, Knights D, Reyes JA, et al. Predictive functional profiling of microbial communities using 16S rRNA marker gene sequences. *Nature biotechnology.* (2013) 31:814-21

18. Segata N, Izard J, Waldron L, Gevers D, Miropolsky L, Garrett WS, et al. Metagenomic biomarker discovery and explanation. *Genome biology.* (2011) 12:1-18
